# Neighborhood Environmental Health and Premature Death From Cardiovascular Disease

**DOI:** 10.5888/pcd15.170220

**Published:** 2018-02-01

**Authors:** Anne H. Gaglioti, Junjun Xu, Latrice Rollins, Peter Baltrus, Laura Kathryn O’Connell, Dexter L. Cooper, Jammie Hopkins, Nisha D. Botchwey, Tabia Henry Akintobi

**Affiliations:** 1 National Center for Primary Care, Morehouse School of Medicine, Atlanta, Georgia; 2 Prevention Research Center, Morehouse School of Medicine, Atlanta, Georgia; 3 Center for Geographic Information Systems, School of City and Regional Planning, Georgia Institute of Technology, Atlanta, Georgia; 4 Transdisciplinary Collaborative Center for Health Disparities Research, Satcher Health Leadership Institute, Morehouse School of Medicine, Atlanta, Georgia

## Abstract

**Introduction:**

Cardiovascular disease (CVD) is the leading cause of death in the United States and disproportionately affects racial/ethnic minority groups. Healthy neighborhood conditions are associated with increased uptake of health behaviors that reduce CVD risk, but minority neighborhoods often have poor food access and poor walkability. This study tested the community-driven hypothesis that poor access to food at the neighborhood level and poor neighborhood walkability are associated with racial disparities in premature deaths from CVD.

**Methods:**

We examined the relationship between neighborhood-level food access and walkability on premature CVD mortality rates at the census tract level for the city of Atlanta using multivariable logistic regression models. We produced maps to illustrate premature CVD mortality, food access, and walkability by census tract for the city.

**Results:**

We found significant racial differences in premature CVD mortality rates and geographic disparities in food access and walkability among census tracts in Atlanta. Improved food access and walkability were associated with reduced overall premature CVD mortality in unadjusted models, but this association did not persist in models adjusted for census tract population composition and poverty. Census tracts with high concentrations of minority populations had higher levels of poor food access, poor walkability, and premature CVD mortality.

**Conclusion:**

This study highlights disparities in premature CVD mortality and neighborhood food access and walkability at the census tract level in the city of Atlanta. Improving food access may have differential effects for subpopulations living in the same area. These results can be used to calibrate neighborhood-level interventions, and they highlight the need to examine race-specific health outcomes.

## Introduction

Cardiovascular disease (CVD) is the leading cause of death for men and women and disproportionately affects racial/ethnic minority groups in the United States and in the state of Georgia (1,2). Public health efforts to reduce CVD mortality focus on increasing equitable access to healthy food and opportunities for physical activity in neighborhoods. Such favorable neighborhood conditions are associated with healthy diet and exercise behaviors and reduced risk for CVD (3–6). Low-income and minority neighborhoods have poor access to healthy food and are less likely to be walkable; these factors may contribute to the well-established racial and socioeconomic disparities in CVD outcomes (7,8).

People living in communities with high levels of health disparities are aware of and concerned about CVD risk and support targeted intervention strategies (9). The Racial and Ethnic Approaches to Community Health (REACH) project, funded by the Centers for Disease Control and Prevention (CDC) and led by The Morehouse School of Medicine Prevention Research Center, conducted a needs assessment in Atlanta in collaboration with community partners to identify the health concerns of communities and residents (10,11). The REACH project uses community based participatory research (CBPR) methods and principles, which means that its work engages community partners in the research process as partners at all stages of the research spectrum (12). Cardiovascular health was a top health concern for residents, who identified neighborhood determinants of cardiovascular health, including “stores without fresh fruits and vegetables,” “access to and knowledge of healthy foods,” and “lack of affordable and healthy food and exercise options” (13). Full qualitative data are presented elsewhere (13). Atlanta is located in Fulton County, which has a food insecurity rating of nearly 20%; the US Department of Agriculture defines food insecurity as “a household-level economic and social condition of limited or uncertain access to adequate food” (14,15).

On the basis of qualitative data gathered from community members who were also subsequently engaged in the CDC REACH project, we designed an analysis of socio-ecologic and epidemiologic data to test the community-driven hypothesis that poor food access and walkability in neighborhoods are associated with increased premature CVD mortality.

## Methods

We used a cross-sectional ecological design to examine the relationship between neighborhood-level food access and walkability and premature CVD mortality rates. Eligible units for this study were the 124 census tracts in Atlanta. The main study outcomes were mortality rates at the census tract level overall and among white and black residents, secondary to premature CVD deaths, from 2010 through 2014. Deaths rates were obtained from the Georgia Department of Public Health. Premature death from CVD was defined as a death due to an underlying cause of CVD occurring between the ages of 35 and 64. CVD was defined by ICD (International Classification of Disease) 10 codes I00–I78 or ICD 9 codes 390–434 (16,17). Census tract mortality rates were calculated by using the number of premature CVD deaths in a census tract among the age- and race-specific population in the census tract obtained from the 2010 census (18). For example, black premature mortality rates were calculated by using CVD deaths of black people aged 35 to 64 in the tract among the population aged 35 to 64. Mortality rates were measured as deaths per 1,000 population. A tract had to have at least 5 premature CVD deaths to be included in the analysis; census tracts with fewer than 5 deaths in any race category were excluded for confidentiality reasons. Premature CVD mortality rate was chosen as a marker for uncontrolled CVD or uncontrolled CVD risk factors.

Independent variables assessed were food access and walkability at the neighborhood planning unit (NPU) level. The food access score was developed by the Georgia Institute of Technology Center for Geographic Information Systems, and the walkability score was obtained by the Center for Geographic Information Systems from Walkscore.com. Atlanta is divided into 25 NPUs (19). Food access and walkability variables were assigned to each census tract on the basis of the NPU where most of the census tract area was located. We chose to look at NPU-level neighborhood variables instead of census tract–level variables because this level of geography is more reflective of residents’ access to food and walking routes. Food access was defined as the percentage of no-vehicle households living beyond a 0.9-mile radius of a food outlet in 2012; a high score indicates poor food access. For example, a score of 50 would indicate that 50% of no-vehicle households in the census tract lived beyond a 0.9-mile radius of a food outlet. The American Community Survey defines a no-vehicle household as a household that has zero passenger vehicles available for use by the household (20). The food access variable was developed and validated by the US Department of Agriculture in a 2009 report to Congress (21). The walkability score measures NPU walkability in 2012 and considers walkability in relationship to amenities, population density, and road metrics; a high score indicates better walkability, and the score ranges from 0 to 100 (22,23). Covariates, taken from the 2010 census, were tract-level age and race distribution (race-specific population in the age categories 35–44, 44–54, and 55–64), percentage of black residents in the census tract, and percentage of the population living below 200% of the federal poverty level. We adjusted for the age distribution of the tract population in the model because the mortality data used in this analysis were not age-adjusted. We merged data sets based on census tract identification number. Frequencies and descriptive statistics were tabulated to characterize the sample. We assessed differences between the census tracts included in the analysis and excluded tracts by using independent sample *t* tests.

Separate linear regression models were constructed to examine the bivariate associations between food access score, walkability score, age distribution, percentage of black residents, and poverty and premature CVD mortality rates at the census tract level. We then constructed multivariate models incrementally to examine changes in β coefficients of the independent variables as covariates were added. We included all variables from the bivariate analysis, because all variables were significantly associated (*P* < .05) with the overall premature CVD mortality rate. Model 1 included the independent variables of census tract–level food and walkability score. Model 2 added the population age distribution in the census tract to Model 1. Model 3 added the percentage of black residents to Model 2. Model 4 added the percentage of the census tract population living below 200% of the federal poverty level to Model 3.

We examined effect modification for census tract poverty and the percentage of black residents with both food access and walkability score in a series of regression models. We also examined characteristics and mortality rates of census tracts by tract poverty level. A 2-tailed level of significance was set at *P* = .05, and all analyses were conducted using SAS version 9.3 (SAS Institute, Inc). We produced maps to illustrate premature CVD mortality, food access, and walkability by census tract by using Arc GIS version 10.5.1 (ESRI). The Morehouse School of Medicine institutional review board approved this study.

## Results

Of Atlanta’s 124 census tracts, 87 tracts met inclusion criteria for overall premature CVD deaths; these tracts contained 73% of the city’s population aged 35 to 64 in 2010 and accounted for a total of 1,225 deaths from 2010 through 2014 and a premature CVD mortality rate of 11 per 1,000. Seventy-one census tracts were included for calculating premature CVD deaths among black residents, accounting for 1,038 deaths over the study period and with a significantly higher mortality rate of 15.6 per 1,000 (*P* < .001) when compared with overall premature CVD mortality rates in the same census tracts (13.8 per 1,000). Only 12 census tracts met inclusion criteria for premature CVD death among white residents; these tracts had 79 deaths and a premature CVD mortality rate of 6 per 1,000.

Black premature CVD deaths accounted for a disproportionate number of the of the 1,225 premature CVD deaths in all census tracts in the city of Atlanta. Nearly 85% (1,038 of 1,225) of premature CVD deaths were among the black population aged 35 to 64, although blacks make up only 52% (80,019 of 153,312) of the city’s total population aged 35 to 64 (Table 1). The tracts we excluded because they did not meet our criteria of a minimum of 5 premature CVD deaths in the study period were home to 42,147 residents aged 35 to 64 (27% of the total Atlanta population aged 35–64), were majority white, had significantly higher (better) mean walkability scores (64.6, standard deviation [SD] 14.7 vs 46.8, SD, 17.5; *P* < .001), and had significantly lower (better) mean food access scores (1.02, SD 2.1 vs 8.0, SD, 7.2; *P* < .001) than census tracts included in the overall premature mortality group. The excluded tracts had a significantly lower percentage of their population living below 200% of the federal poverty level when compared with the tracts that met inclusion criteria for the study (29.6% vs 51.7%) (Table 1).

**Table 1:** Characteristics of Census Tracts with Premature Deaths From Cardiovascular Disease (CVD), by Race, Atlanta, Georgia, 2010–2014^a^

Characteristics	Overall Premature CVD Mortality Rate, 87 Tracts	Black Premature CVD Mortality Rate, 71 Tracts	White Premature CVD Mortality Rate, 12 Tracts	All City Tracts, 124 Tracts	Excluded Tracts, 37 Tracts^b^
**Race-specific mortality rate (deaths per 1,000 population)**	12.03 (6.10)	15.57 (5.55)	10.81 (16.56)	NA	NA
**Food access score^c^**	7.96 (7.21)	9.04 (7.24)	1.18 (1.50)	5.89 (6.91)	1.02 (2.10)^e^
**Walkability score^d^**	46.79 (17.53)	45.76 (17.60)	58.75 (17.24)	52.09 (18.57)	64.56 (14.70)^e^
**Total census tract population below 200% of federal poverty level**	51.70 (22.15)	57.79 (17.44)	25.19 (21.30)	45.05 (24.28)	29.58 (22.07)^e^
**Total black census tract population**	71.76 (30.25)	82.10 (20.52)	21.16 (20.73)	57.48 (36.80)	23.92 (28.14)^e^
**No. premature CVD deaths**					
Overall	1,225	1,116	122	1,225	NA
White residents	79	18	79	79	NA
Black residents	1,038	1038	34	1038	NA
**Distribution of residents aged 35 to 64**					
White residents	31,081	11,103	15,916	61,840	30759
Black residents	72,900	67,920	4,302	80,019	7,119
Total	111,165	83,344	22,596	153,312	42,147

a Population and demographic data were obtained from the 2010 census. Early CVD deaths by racial subgroup were obtained from the Georgia Department of Health for years 2010–2014. Values are mean (standard deviation) unless otherwise indicated.

b Excluded because these tracts had fewer than 5 CVD deaths.

c Percentage of zero-vehicle households living beyond a 0.9-mile radius of a food outlet. Food access score was obtained from the Georgia Institute of Technology Center for Geographic Information Systems and is based on 2012 data. Scale for food access scores ranges from 0% to 100%; lower scores indicate better food access.

d Walkabilty score was obtained from Walkscore.com and is based on 2012 data. Scale for walkability scores ranges from 0 to 100; higher scores indicate better neighborhood walkability.

e Significantly different from the 87 tracts based on *t* test *P* < .001.

The interaction effect for food access and poverty was found to be significant (*P* = .01) in a model including food access, percentage poverty, and an interaction term for food availability score and percentage population below 200% poverty level (Appendix Table 1). However, the interaction term was no longer significant after the percentage of black population was added to the model. No other interaction terms in any models were significant. High-poverty tracts have a significantly higher percentage black population, a significantly higher premature CVD mortality rate, and higher proportion of residents living below 200% of the federal poverty level. We found no significant difference in walkability score between high-poverty and low-poverty census tracts (Appendix Table 2).

Census tract food access score, walkability score, percentage population in each 10-year age category between age 35 and 64, percentage black population, and percentage living below 200% of the poverty level were significant in the univariate analysis and were included the adjusted models (Table 2).

**Table 2:** Bivariate Models of Census Tract (N = 158) Characteristics and Cardiovascular Disease (CVD) Mortality Rates, Atlanta, Georgia, 2010–2014^a^

Characteristics	Overall Premature CVD Mortality Rate, β (95% CI), 87 Tracts	*P* Value	Black Premature CVD Mortality Rate, β (95% CI), 71 Tracts	*P* Value
Food access score^b^	0.40 (0.25 to 0.56)	<.001	0.12 (−0.06 to 0.29)	.20
Walkability score^c^	−0.11 (−0.18 to −0.04)	.02	−0.04 (−0.12 to 0.03)	.23
Percentage residents with income below 200% of poverty line	0.16 (0.11 to 0.21)	<.001	0.03 (−0.05 to 0.10)	.48
Percentage black residents	0.15 (0.12 to 0.18)	<.001	0.03 (−0.04 to 0.09)	.43
Age, y^d^				
35–44	−0.87 (−1.11 to −0.64)	<.001	−0.89 (−1.40 to −0.38)	.006
45–54	−0.21 (−0.63 to 0.21)	.31	−0.32 (−0.64 to 0.00)	.05
55–64	0.46 (0.02 to 0.91)	.04	−0.01 (−0.46 to 0.43)	.95

Abbreviation: CI, confidence interval.

a Population and demographic data were obtained from the 2010 census. Early CVD deaths by racial subgroup were obtained from the Georgia Department of Health for years 2010–2014.

b Percentage of zero-vehicle households living beyond a 0.9-mile radius of a food outlet. Food access score was obtained from the Georgia Institute of Technology Center for Geographic Information Systems and is based on 2012 data. Scale for food access scores ranges from 0% to 100%; lower scores indicate better food access.

c Walkability score was obtained from Walkscore.com and is based on 2012 data. Scale for walkability scores ranges from 0 to 100; higher scores indicate better neighborhood walkability.

d For overall CVD mortality tracts, all race categories in the census tract were used to calculate age groups; for black premature CVD mortality rate, only the black population in the census tract was used to calculate age groups.

In Model 1, poor food (β, 0.36; 95% confidence interval [CI]; 0.20–0.52; *P* < .001) and walkability scores (β, −0.07, 95% CI; −0.14 to −0.01; *P* < .05) were significantly associated with higher overall premature CVD mortality rates (Table 3). However, these associations were not significant in the model of the group of tracts that included only black premature CVD mortality rates. In Model 2, which adds the population distributions in 10-year categories for residents aged 35 to 64 in the tract, the poor food access score (β, 0.23; CI, 0.06–0.39; *P* < .01) was significantly associated with a high overall premature CVD mortality rate, and a high proportion of the population aged 35 to 44 was associated with low overall premature CVD mortality rate at the census tract level. However, the walkability score no longer had a significant association with overall premature CVD mortality rate after addition of the age distribution variables. In Model 3, which adds the percentage black population in the census tract, and Model 4, which includes the percentage black population and the percentage population living below 200% of the poverty level, a high percentage black population in the census tract (Model 3: β, 0.12; 95% CI, 0.08–0.17; *P* < .001; Model 4: β, 0.11; 95% CI, 0.06–0.17; *P* < .001) was significantly associated with high overall premature CVD mortality rates. Neither the food nor the walkability scores were significantly associated with overall or black premature CVD mortality rates in these models. Multivariable models for white-race tracts were not constructed because of small numbers.

**Table 3:** Multivariable Models of Characteristics and Overall and Black Premature Cardiovascular Disease (CVD) Mortality Rates in Census Tracts (N = 158), Atlanta, Georgia, 2010–2014^a^

Characteristics	Overall Premature CVD Mortality Rate in Census Tracts, 87 Tracts, β (95% CI)	*P* Value	Black Premature CVD Mortality Rate in Census Tracts, 71 Tracts, β (95% CI)	*P* Value
**Model 1: Food and walk scores**				
Food access score^b^	0.36 (0.20 to 0.52)	<.001	0.10 (−0.08 to 0.28)	.29
Walkability score^c^	−0.07 (−0.14 to −0.01)	.03	−0.04 (−0.11 to 0.04)	.33
**Model 2: Food and walkability scores + age categories^b^**				
Food access score^b^	0.23 (0.06 to 0.39)	.004	0.03 (−0.15 to 0.21)	.77
Walkability score **c**	−0.02 (−0.09 to 0.04)	.46	0.02 (−0.06 to 0.10)	.58
Population aged 35–44^d^	−0.63 to (−0.95 to −0.32)	<.001	−1.01 (−1.77 to −0.25)	.009
Population aged 45–54^d^	−0.08 (−0.54 to 0.39)	.74	−0.02 (−0.50 to 0.45)	.92
Population aged 55–64^d^	0.15 (−0.39 to 0.69)	.57	0.26 (−0.23 to 0.75)	.30
**Model 3: Food and walkability scores + age + black population**				
Food access score^b^	0.08 (−0.07 to 0.22)	.29	0.09 (−0.10 to 0.28)	.36
Walkability score^c^	0.03 (−0.03 to 0.09)	.30	−0.01 (−0.10 to 0.08)	.78
Population aged 35–44^d^	−0.15 (−0.47. 0.16)	.34	−1.29 (−2.10 to −0.47)	.002
Population aged 45–54^d^	−0.39 (−0.79 to 0.00)	.05	0.06 (−0.41 to 0.53)	.81
Population aged 55–64^d^	0.35 (−0.10 to 0.79)	.12	0.24 (−0.24 to 0.72)	.33
Percentage black population	0.12 (0.08 to 0.17)	<.001	−0.07 (−0.16 to 0.01)	.09
**Model 4: Food and walkability scores + age + black population + poverty**				
Food access score^b^	0.05 (−0.10 to 0.21)	.51	0.12 (−0.09 to 0.32)	.26
Walkability score^c^	0.03 (−0.03 to 0.09)	.26	−0.00 (−0.09 to 0.09)	.97
Population aged 35–44^d^	−0.19 (−0.54 to 0.16)	.29	−1.38 (−2.22 to −0.53)	.001
Population aged 45–54^d^	−0.48 (−0.91 to −0.06)	.03	0.09 (−0.40 to 0.56)	.74
Population aged 55–64^d^	0.47 (−0.03 to 0.97)	.06	0.18 (−0.34 to 0.69)	.50
Percentage black residents	0.11 (0.06 to 0.17)	<.001	−0.07 (−0.15 to 0.02)	.15
Percentage with incomes below 200% poverty level	0.02 (−0.05 to 0.09)	.59	−0.04 (−0.14 to 0.06)	.48

Abbreviation: CI, confidence interval.

a Population and demographic data were obtained from the 2010 census. Early cardiovascular disease deaths by racial subgroup were obtained from the Georgia Department of Health for years 2010–2014. Food access score was obtained from the Georgia Institute of Technology Center for Geographic Information Systems and is based on 2012 data. Walkability score was obtained from Walkscore.com and is based on 2012 data.

b Percentage of zero-vehicle households living beyond a 0.9-mile radius of a food outlet. Food access score was obtained from the Georgia Institute of Technology Center for Geographic Information Systems and is based on 2012 data. Scale for food access scores ranges from 0% to 100%; lower scores indicate better food access.

c Walkability score was obtained from Walkscore.com and is based on 2012 data (23). Scale for walkability scores ranges from 0 to 100; higher scores indicate better neighborhood walkability.

d For overall CVD mortality tracts, all race categories in the census tract were used to calculate age groups, for black premature CVD mortality rate, only the black population in the census tract was used to calculate age groups.

**Figure 1:**
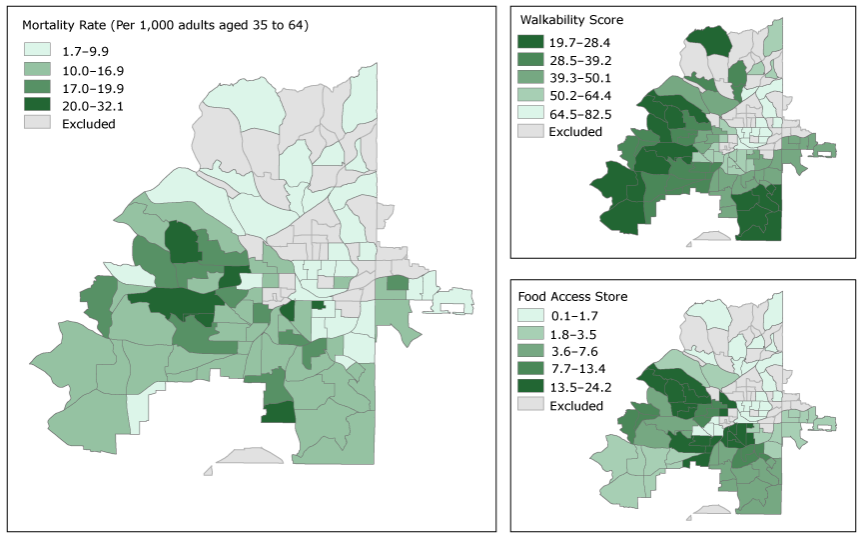
Overall premature cardiovascular disease (CVD) mortality rate, walkability score, and food access score by census tract, Atlanta, Georgia, 2010–2014. Walkability score is on a scale of 0 to 100, and a higher walkability score indicates worse walkability. Food access scores range from 0 to 100, and a low score indicates better food access. Food access scores and walkability scores are presented in quintiles; these categories are common to both Figure 1 and Figure 2 so the maps can be directly compared.

**Figure 2:**
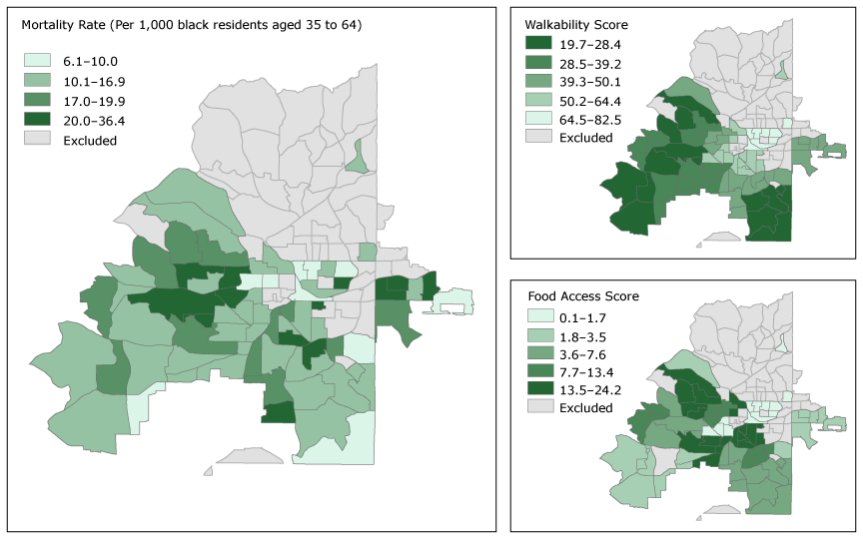
Premature cardiovascular disease (CVD) mortality rate, walkability score, and food access score among black residents, Atlanta, Georgia, 2010–2014. Walkability score is on a scale of 0 to 100, and a higher walkability score indicates worse walkability. Food access scores range from 0 to 100, and a low score indicates better food access. Premature CVD mortality rates are presented in quintiles; these mortality rate categories are common to both Figure 1 and Figure 2 so the maps can be directly compared. Food access scores and walkability scores are presented in quintiles; these categories are also common to both Figure 1 and Figure 2 so the maps can be directly compared.

Maps of food and walkability scores and maps of mortality for overall premature CVD tracts (Figure 1) and black-race premature CVD mortality rate tracts (Figure 2) show that for overall premature CVD mortality rate tracts, the highest mortality rates occur in the southwestern quadrant of the city. This pattern is similar to the pattern for food access, and, to a lesser extent, walkability. These geographic patterns also appear in the premature CVD mortality census tract maps for the black population.

## Discussion

We found significant racial differences in premature CVD mortality rates and disparities in neighborhood-level characteristics of food access and walkability in this cross-sectional ecologic study. We also found that healthy socio-ecologic characteristics of neighborhoods, such as food access, have different effects on race-specific premature CVD mortality rates at the census tract level. However, when models were fully adjusted for neighborhood poverty and the percentage black population, better neighborhood food access was not significantly associated with premature CVD mortality. We found marked disparities in the population composition and socio-ecologic characteristics of residents of census tracts in Atlanta that were included in this study because they met a threshold number of premature CVD deaths during the study period compared with tracts that were not included. The excluded tracts, where premature CVD deaths were rare, had significantly better food access, walkability, lower poverty rates, and fewer minority residents. The census tracts that did meet criteria for inclusion, which had higher mortality from premature CVD, had majority black populations and had higher burdens of poverty, poor food access, and poor walkability. The covariate that was significantly associated with overall premature CVD mortality at the census tract level in the fully adjusted multivariable model was the percentage of the census tract population that was of black race.

Studies have found a positive association between neighborhood food access and walkability and CVD risk factors (24,25). However, our outcome variable of premature CVD has not been previously tested; outcomes that have been studied include body mass index (weight in kilograms divided by height in square meters), type 2 diabetes, prediabetes, hypertension, and lipid disorders. Although these outcomes can contribute to a risk profile for premature CVD death, disparities in access to optimal treatment or prevention for black populations may account for our findings. Additionally, our study did not find an association between neighborhood walkability and premature CVD mortality, although previous studies found an association with neighborhood walkability, increased physical activity and reduced obesity (3–6). Although these associations may have reduced risk factors for premature CVD death, other factors such as disparities in access to and uptake of medical treatment may account for the lack of association noted here. Although the walkability score used in this study has been shown to be highly predictive of how people walk for nonwork purposes such as shopping or school travel, these patterns differ among socioeconomic subgroups (23). Additionally, living in a healthy place can affect different subpopulations in different ways (26). For example, modification of CVD risk factors may have less effect among those with diagnosed or advanced CVD when compared with those who are yet undiagnosed and have modifiable risk factors, such as obesity or inactivity (27). The different relationship between neighborhood-level food access and premature CVD mortality in the 2 series of models presented here supports the importance of examining how the characteristics of a place might have different effects on the health of the population living there. For example, studies such as ours could be used to direct and support interventions that would potentially have the most impact on improving health outcomes or reducing health disparities based on the demographics of the subpopulations living in an area. Well-documented racial health disparities in CVD burden and outcomes may also account for our findings. Black people have higher burdens of CVD at younger ages when compared with other races/ethnicities, and significant disparities are well-documented in treatment, diagnosis, access to care, and risk factors for CVD (7,8).

Future studies should include replication of this work in other cities and exploration of other neighborhood-level variables that might differentially affect racial subgroups. Such work will enable projects that improve the neighborhood built environment to maximize health impact based on the demographic composition of the population. Information from studies such as ours can be used to engage community members to further interpret these findings, leading to development of intervention strategies with community stakeholders that address neighborhood health disparities.

This study has several limitations. This small study of one major metropolitan area may not be generalizable to other metropolitan areas or to nonmetropolitan areas. Mortality rates were not age–sex adjusted, although we did attempt to age-adjust in the multivariable models by accounting for the age distribution in the census tract. This study examined aggregated mortality rates over a 5-year period, an approach necessary to maintain confidentiality given the small geographic unit of analysis. Aggregating mortality rates over a 5-year period allowed us to conduct this small-area analysis but negated the ability to account for individual-level characteristics of premature CVD deaths, including sex, income, education level, and presence of CVD risk factors such as obesity and hypertension. Walkability was measured using Walk Score (22), which does not measure the quality of the pedestrian environment. Other measures that better characterize space that could be used for exercise, such as parks and walking trails, could be used in future work.

The strengths of this study are its potential to engage community stakeholders to address the contexts and systems associated with premature CVD. This CBPR study was the result of a partnership with Atlanta neighborhood residents who identified that limited access to healthy foods and opportunities to get physical activity were determinants of the community burden of CVD. CBPR was intentionally used because of its emphasis on community–academic partnerships with shared leadership in the planning, implementation, evaluation, and dissemination of interventions (12). The REACH Initiative was designed to improve access to quality health care and reduce risk factors for diabetes and CVD among black residents in Atlanta, Georgia, through several mechanisms: 1) making streets safe and walkable for children, which emerged as an important priority toward increased opportunities for safe physical activity; 2) The Healthy Corner Store Initiative, which was designed through strategic partnership with local corner stores to increase access to healthy food options (28); and 3) through employing community health workers who support residents to prevent and manage CVD risk factors.

In 2008, the National Institutes of Health highlighted CBPR as a core strategy in eliminating disparities (29). This CBPR approach, coupling secondary, geographically relevant data with neighborhood health priorities, has elevated community assessment to more than an exercise of cataloging health disparities to one that serves to democratize data toward targeting approaches that are contextually responsive in reducing premature CVD.

This study highlights racial disparities in premature CVD mortality and neighborhood socio-ecologic characteristics of food access and walkability at the census tract level in the city of Atlanta. It demonstrates that improving food access at the neighborhood level may affect different subpopulations living in an area differently, and that although neighborhood food access and walkability may positively affect CVD risk factors, these effects may not translate to reductions in racial disparities in premature CVD mortality. These results can be used to calibrate neighborhood-level interventions based on racial/ethnic or other demographic characteristics and highlight the need to examine racially stratified health outcomes. Finally, motivating hypothesis testing with secondary data by using the lived experience of community members allows results to be easily translated to and interpreted by community residents. This can result in increased engagement and sustainability of future intervention strategies.

## Appendix

**Table 1:** Tests for Effect Modifiers, Census Tracts (N = 158) with Premature Cardiovascular (CVD) Deaths, Atlanta, Georgia 2010–2014

Effect Modifier	Overall Premature CVD Mortality Rate, 87 Tracts, *P* Value	Black Premature CVD Mortality Rate, 71 Tracts, *P* Value
Section 1: Food × effect modifier		
Food × percentage below 200% poverty	.01	.60
Food × percentage black population	.70	.42
Section 2: Walk × effect modifier		
Walk × percentage below 200% poverty	.52	.76
Walk × percentage black population	.55	.70
Section 3: Food and walk × poverty		
Food × percentage below 200% poverty	.01	.78
Walk × percentage below 200% poverty	.78	.67
Section 4: Food and walk × percentage black population		
Food × percentage black population	.75	.28
Walk × percentage black population	.63	.49
Section 5: Food and walk × effect modifiers all together		
Food × percentage below 200% poverty	.41	.79
Food × percentage black population	.35	.25
Walk × percentage below 200% poverty	.92	.90
Walk × percentage black population	.60	.54

**Table 2:** Characteristics of High-Poverty Versus Low-Poverty Census Tracts (N = 87) with Premature Cardiovascular Disease (CVD) Deaths, Atlanta, Georgia 2010–2014

Characteristic	High-Poverty Tracts (n = 43)	Low-Poverty Tracts (n = 44)	*P* Value
Census tract, mean (standard deviation)			
Race-specific mortality rate (deaths per 1,000 population)	15.0 (5.4)	9.1 (5.3)	<.001
Food access score^a^	12.2 (7.1)	3.8 (4.3)	<.001
Walkability score^b^	44.8 (16.2)	48.7 (18.7)	.30
Percentage of total population below 200% poverty level	70.5 (9.8)	32.9 (13.1)	<.001
Percentage of total black population	89.5 (9.0)	54.4 (33.6)	<.001
Census tract population, no.			
Total premature CVD deaths	596	629	—
White premature CVD Deaths	6	73	—
Black premature CVD Deaths	554	484	—
White population aged 35–64^c^	2,336	28,745	—
Black population aged 35–64^c^	35,284	37,616	—
Total population aged 35-64^c^	39,638	71,527	—

Abbreviation: —, not applicable.

^a^ Percentage of zero-vehicle households living beyond a 0.9-mile radius of a food outlet. Food access score was obtained from the Georgia Institute of Technology Center for Geographic Information Systems and is based on 2012 data. Scale for food access scores ranges from 0% to 100%; lower scores indicate better food access.

^b^ Walkability score was obtained from Walkscore.com and is based on 2012 data (23). Scale for walkability scores ranges from 0 to 100; higher scores indicate better neighborhood walkability.

^c^ For overall CVD mortality tracts, all race categories in the census tract were used to calculate age groups. For black premature CVD mortality rate, only the black population in the census tract was used to calculate age groups. For white premature CVD mortality rate, only the white population in the census tract was used to calculate age groups.
